# Gecko-inspired self-adhesive packaging for strain-free temperature sensing based on optical fibre Bragg gratings

**DOI:** 10.1038/s41598-023-30949-6

**Published:** 2023-03-13

**Authors:** Shuochao Liu, Pingyu Zhu, Fumin Xie, Marcelo A. Soto

**Affiliations:** 1 School of Mechanical and Electrical Engineering, Guangzhou University, 510056 Guangzhou, People’s Republic of China; 2 Department of Electronics Engineering, Universidad Técnica Federico Santa María, 2390123 Valparaíso, Chile

**Keywords:** Optical sensors, Fibre optics and optical communications

## Abstract

The large development of fibre Bragg gratings (FBGs) over decades has made this kind of structures one of the most mature optical fibre sensing technologies existing today, demonstrating key features for a very wide range of applications. FBG sensors are fragile and must be normally protected for real-field applications, although challenging packaging designs are required to mitigate temperature-strain cross-sensitivity issues. Here, a polydimethylsiloxane (PDMS) packaging with a microarray structure that provides gecko-inspired dry adhesion is proposed for strain-free FBG-based temperature sensing. Besides offering protection, the PDMS packaging with an embedded polyamide capillary damps the mechanical strain transferred to the optical fibre, providing FBG-based temperature sensing with a negligible impact of strain. In addition, the microarray structure imprinted on one surface of the packaging provides gecko-inspired dry adhesion based on van der Waals forces. This feature enables the packaged optical fibre sensor to be attached and detached dynamically to nearly any kind of smooth surface, leaving no residuals in the monitored structure. Experimental results verify a fast and accurate temperature response of the sensor with highly mitigated impact of residual strain. The proposed packaged sensor can be used in application where glue is not allowed nor recommendable to be used.

## Introduction

Fibre Bragg gratings (FBGs) are widely used for sensing applications in a broad range of fields due to their multiple advantages such as lightweight, small size, fast response, immunity to electromagnetic interference, high sensitivity, and high reliability even in harsh operating scenarios^1,2^. FBG sensors inscribed in bare optical fibres are fragile and they normally require to be carefully packaged and glued on the structures to be monitored^3–5^. The use of packaging can actually protect embedded FBG sensors, however, the packaging design turns out to be challenging if issues with the cross-sensitivity between strain and temperature want to be mitigated. For instance, a proper packaging design is required to reduce strain transfer and allow for strain-free temperature sensing^6^. Unfortunately, the large dimensions required for the packaging structure makes this kind of solution unsuitable for some applications, where small, non-invasive sensors are required. In addition, the use of glue to paste bare or packaged FBGs could pollute the surface of monitored structures. This is highly undesired in many real applications, as for instance for monitoring microelectronic or chip devices^7,8^. While the effects of different kinds of glues for FBG sensing have been investigated in the literature^9–11^, in all cases the strain transfer provided by the glue would affect temperature sensing, making it difficult to reach strain-free thermal monitoring. On the other hand, removing glued FBG sensors from a structure might result in adhesive residuals that remain in the structure; this is normally unwanted in many cases. Therefore, the development of an innovative FBG packaging and gluing method that leaves no adhesive residuals in the monitored surface could be of great interest for many practical applications.

For over two decades, researchers have been investigating alternative bioinspired solutions to develop dry adhesives for a large number of applications^12–16^. In particular, the strong footpads of Tokay geckos have inspired researchers to develop an impressive and diverse set of artificial dry adhesives^13^. These dry adhesives are based on the van der Waals force used by geckos to climb walls^17,18^. In this case however, this adhesive force is obtained by designing and manufacturing micro or nano-scale synthetic structures. This way, by mimicking the geometric features of gecko toe pad structures, and thanks to significant recent progress in micro- and nanofabrication techniques, these gecko-inspired dry adhesives have demonstrated similar or even larger adhesive forces compared to real gecko footpads^12^.

In this paper, a packaging structure with a microstructure array is proposed to protect FBG sensors, while providing gecko-inspired dry adhesive capabilities through van der Waals force. Using a material with low elastic modulus, like polydimethylsiloxane (PDMS), and an embedded polyamide (PI) capillary, the packaging provides a very low strain transfer between the monitored object and the FBG sensor, enabling strain-free temperature sensing capabilities. With no need of glue, the self-adhesive sensing structure can also be easily and dynamically attached and detached from a given monitored object. This gecko-inspired microarray structure not only shows a dry adhesive effect, but also can maintain the close contact between layers while releasing the interlaminar strain. Experimental results demonstrate the capability of the packaged FBG sensors to be attached to surfaces made of different materials and to be detached leaving no residuals. The tangential adhesion force provided by the packaging to four different materials is experimentally evaluated. In addition, the thermal response of the embedded FBG sensor is evaluated, along with the verification of the high strain mitigation provided by the packaging. Results also demonstrate that the use of the embedded PI capillary provides additional strain mitigation compared to the use of simple PDMS packaging^19^ without capillary. The proposed self-adhesive packaging provides a new feature to FBG sensing, permitting the sensors to be easily removed and reused in several applications. It is also expected that the proposed packaging to have great impact for strain-free temperature monitoring of structures or devices where glue is not possible to be used.

## Working principle and fabrication process

### Fibre Bragg grating sensors

FBGs are optical structures made by periodical changes of the refractive index of the optical fibre core^1,2^. The axial period of the grating defines a resonance wavelength, known as Bragg wavelength, for which incoming light is reflected in phase, while all other wavelengths are transmitted through. The Bragg wavelength ${\uplambda }_{B}=2{n}_{eff}\Lambda$ depends on the effective core refractive index ${n}_{eff}$ and the period $\Lambda$ of the grating. These two parameters depend on the fibre temperature and strain, and therefore if any of these environmental variables change, the FBG reflection spectrum, centred at ${\uplambda }_{B}$, turns out to be shifted. The relative frequency shift can be written as^1,2^:

1 $$\frac{\Delta {\lambda }_{B}}{{\lambda }_{B}}={k}_{\varepsilon }\Delta \varepsilon +{k}_{T}\Delta T$$

where $\Delta {\lambda }_{B}$ is the Bragg wavelength shift, ${\lambda }_{B}$ is the initial central wavelength of the FBG, ${k}_{\varepsilon }$ and ${k}_{T}$ are the strain and temperature coefficients of the FBG, respectively; and $\Delta \varepsilon$ and $\Delta T$ are the changes in the external fibre strain and temperature, respectively. Based on this principle, FBGs can be used for measuring temperature and axial strain variations for a very broad range of applications.

Note that, according to Eq. (1), the Bragg wavelength depends on both strain and temperature changes, making it impossible to distinguish between each other. To provide strain-free temperature sensing, the FBG sensor must be attached to the monitored structure using a method that secures low strain-transfer efficiency. This low strain-transfer efficiency is in this work achieved by packaging the FBG with a structure made of a soft material, i.e., a material with low Young modulus, which highly damps the strain transferred to the FBG. Further strain isolation, without negative impact on the thermal transfer, can also be achieved by inserting the embedded optical fibre into a polyamide capillary, as will be detailed hereafter.

### Mitigation of strain transfer

**Figure 1:**
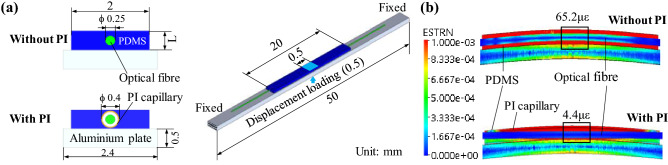
Simulations based on finite element method to analyse the strain transferred from an aluminium plate to the packaged FBG sensor. (**a**) FEM model with dimensions of the simulated structures. (**b**) Results indicating the strain distribution when a vertical displacement of 0.5 mm is applied to the centre of the plate. The colours represent the strain obtained by the simulations in strain (ε) units.

To reduce the strain that is transferred from the monitored structure to the FBG sensor, a PDMS packaging is proposed here with an embedded PI capillary, where the optical fibre containing an FBG sensor is inserted. To better analyse the strain reduction process, simulations through a three-dimensional finite element method (3D-FEM) are first presented. For this, a constant displacement of 0.5 mm is applied to an aluminium plate with a 20 mm × 2 mm PDMS-packaged FBG sensor on top, as shown in Fig. 1a. The diameter of the optical fibre with coating is 0.25 mm, while the outer and inner diameters of the PI capillary are 0.46 mm and 0.4 mm, respectively, thus allowing some gap between the fibre and capillary to help for the strain mitigation. The thickness of the PDMS packaging is 0.5 mm.

The FEM model is solved using the parameters indicated in Table 1. Figure 1b shows the FEM results for the case of using no PI capillary (top image) and when using capillary (bottom image). Results point out that, whilst the applied displacement induces a surface strain of 782.1 με in the aluminium plate, the PDMS packaging with no capillary reduces the strain transferred to the FBG sensor to 65.2 με, representing only the 8.3% of the strain induced in the aluminium plate. In addition, the transferred strain is further reduced to 4.4 με when the PI capillary is used, corresponding to the 0.56% of the strain in the plate. The large strain reduction can be better understood by analysing the strain distribution observed in the FEM results. It is possible to observe that the PDMS packaging experiences large strain, so that the lowest section of the packaging (i.e., the part facing the aluminium plate) can easily deform and absorb the strain thanks to its low Young modulus, providing a buffer layer that keeps the embedded optical fibre with minimum strain. In addition, when using the PI capillary, the small gap between capillary and fibre permits the fibre to bend less than the packaging, further mitigating the strain transferred to the FBG sensor.

**Table 1:** Parameters used in the 3D-FEM-based simulations.

Material	Young modulus (MPa)	Poisson's ratio
Optical fibre	72,000	0.20
PDMS	2	0.49
PI capillary	400	0.35
Aluminium plate	69,000	0.33

**Figure 2:**
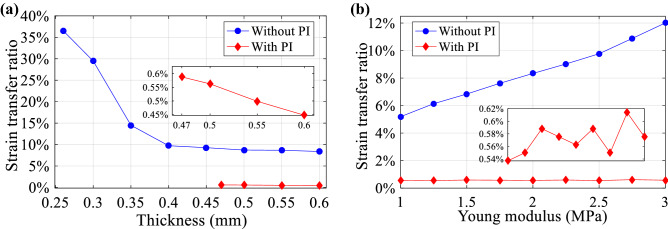
Impact of (**a**) thickness and (**b**) Young modulus of PDMS packaging on strain transfer ratio (ratio between the embedded FBG strain and the strain applied to the PDMS packaging). Comparison between the use of only PDMS without PI capillary (blue curves) and the use of PI capillary embedded into the PDMS packaging (red curves). Inset figures represent a magnified view of the results obtained when using the PI capillary (red curves).

The impact of the packaging on the strain reduction is further analysed by independently modifying the thickness and Young modulus of the PDMS packaging. Figure 2a shows that the increase of the PDMS thickness enhances the strain isolation. Results also indicate that when no PI capillary is used, the PDMS packaging is preferred to have a thickness larger than 0.4 mm, resulting in a strain transfer of about 8%. When the PI capillary is used, the strain transfer remains practically constant around 0.55% for all simulated thickness scenarios. Note that in this case the minimum PDMS thickness is 0.47 mm, which is only 0.01 mm thicker than the outer diameter of the PI capillary. On the other hand, Fig. 2b shows that changes in the packaging Young modulus lead to variations of a few percent in the strain transfer when no PI capillary is used, while there is almost no impact when the capillary is inserted. This could be explained because in the first case the strain damping effect is only determined by the strain absorption of the PDMS packaging, while in the second case the PI capillary has a relevant contribution to the strain reduction.

Based on these results, a packaging with a thickness of 0.5 mm and a Young modulus of 2 MPa is manufactured, as described here below, having a small periodic microstructure to provide self-adhesive features. Note that this microstructure has a depth in the sub-micrometre scale, resulting in negligible impact on the strain transfer simulated here.

### Van der Waals force and dry adhesion

Over the last decade gecko-inspired dry adhesives have been getting a great deal of attention. They are essentially based on mimicking the nano- and micro-structures existing in gecko’s feet, which can dynamically attach to different materials using van der Waals force^12–16^. This type of force results in an attraction between molecules, generated when the gap between the contact interfaces of two objects is very small^20^.

Gecko-inspired synthetic dry adhesives require a precise fabrication of multiscale, hierarchical structures with controlled geometries^12^. Many different geometries and hierarchies of gecko-inspired structures have been developed and fabricated in the last years^12,13^. These kinds of synthetic adhesives try to maintain some important functional properties of the gecko adhesive, such as^16^ (a) being directional, (b) strong attachment with minimal preload, (c) quick and easy detachment from structures, (d) capability to stick to many different materials, (e) clean adhesive leaving no residuals, (f) self-adhesive, (g) non-sticky by default, and (h) having the ability to sustain high levels of adhesion while sliding.

In this work, a periodic wedge-shaped microarray structure is manufactured in the PDMS packaging with an embedded FBG sensor using a diffraction grating. Note that different geometries change the range and degree of force versus separation. In this case, a simple quasi-planar jagged structure with small grooves and periodicity of 10 μm has been designed. When the prepared flexible body surface contacts with other objects, these regular serrated microarrays generate intermolecular van der Waals forces^20^, such as dispersion force and induction force, allowing for the expected gecko-inspired dry adhesion force between the PDMS packaging and different materials, as it will be demonstrated in the “Results and discussion” section.

### Manufacturing process

**Figure 3:**
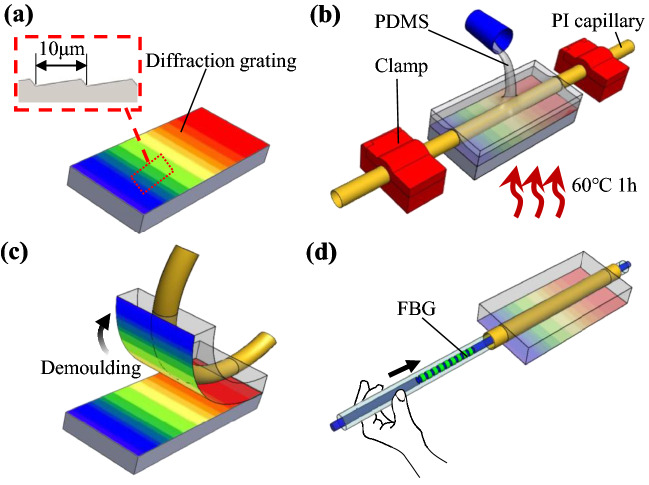
Steps followed to fabricate the self-adhesive PDMS-packaged FBG sensor. (**a**) Use of diffraction grating to imprint a wedge-shaped microarray structure with 10 μm period and 0.343 μm depth. (**b**) PDMS preparation in a mould placed inside a temperature chamber, with a polyamide capillary clamped at both ends. (**c**) Fabricated PDMS packaging is removed from the mould. (**d**) Optical fibre is passed through the polyamide capillary so as the FBG sensor remains loosed in the middle of the packaging.

The packaging proposed in this work is made of PDMS with a microarray adhesive structure on one of the surfaces. In addition, a polyamide (PI) capillary is placed in the middle of the packaging, where the FBG sensor is inserted to remain loose inside. This microarray structure allows one face of the PDMS packaging to strongly attach to the object or structure to be monitored with no need of additional glue. The microarray adhesive structure must be designed to guarantee that a large enough van der Waals force is produced and secure that the PDMS packaging remains well attached to the monitored structure. This microarray structure is fabricated by using a diffraction grating as a fabrication mould. The manufacturing steps are depicted in Fig. 3, and summarised as follows:

1. A diffraction grating with a period of 10 μm is placed as a base plate to be used as a mould, as shown in Fig. 3a. A polyamide capillary is placed over the diffraction grating and clamped at both ends to prevent it from moving and bending during fabrication. The outer and inner diameters of the PI capillary are similar to the FEM simulations, i.e. 0.46 mm and 0.4 mm, respectively.
2. PDMS is prepared following a 10:1 ratio between the solution and curing agent. The prepared PDMS solution is poured into the mould, as shown in Fig. 3b, and left for 1 h to defoam naturally. Then, the mould with PDMS is placed into a temperature chamber at 60ºC for 1 h until the PDMS solidifies.
3. The mould is then taken out from the temperature chamber and the self-adhesive PDMS packaging layer is demoulded, as depicted in Fig. 3c.
4. Finally, the optical fibre is passed through the embedded PI capillary, so that the FBG sensor remains loosely located inside and in the middle of the PI capillary (Fig. 3d). This step completes the fabrication of the self-adhesive packaged FBG sensing structure.

The most critical aspects of the PDMS manufacturing process include the control of three parameters: first, the ratio of curing agent to solution (1:10) to reach a Young modulus of 2 MPa (as analysed by the FEM simulations); second, the curing temperature (60 ºC); and finally, the thickness of the PDMS mixed solution in the mould (0.5 mm). These three factors determine whether the PDMS can be well cured, obtain the required elastic modulus, and demould from the mould to secure the generation of microarray surface structure.

Note that, for comparison purposes, another sample but without PI capillary is also manufactured for comparison. In this case, the optical fibre and FBG sensor are placed over the diffraction grating in step (1). In addition, a small pulling force must be applied from both ends of the fibre to avoid any bending of the FBG sensor and optical fibre during manufacturing.

**Figure 4:**
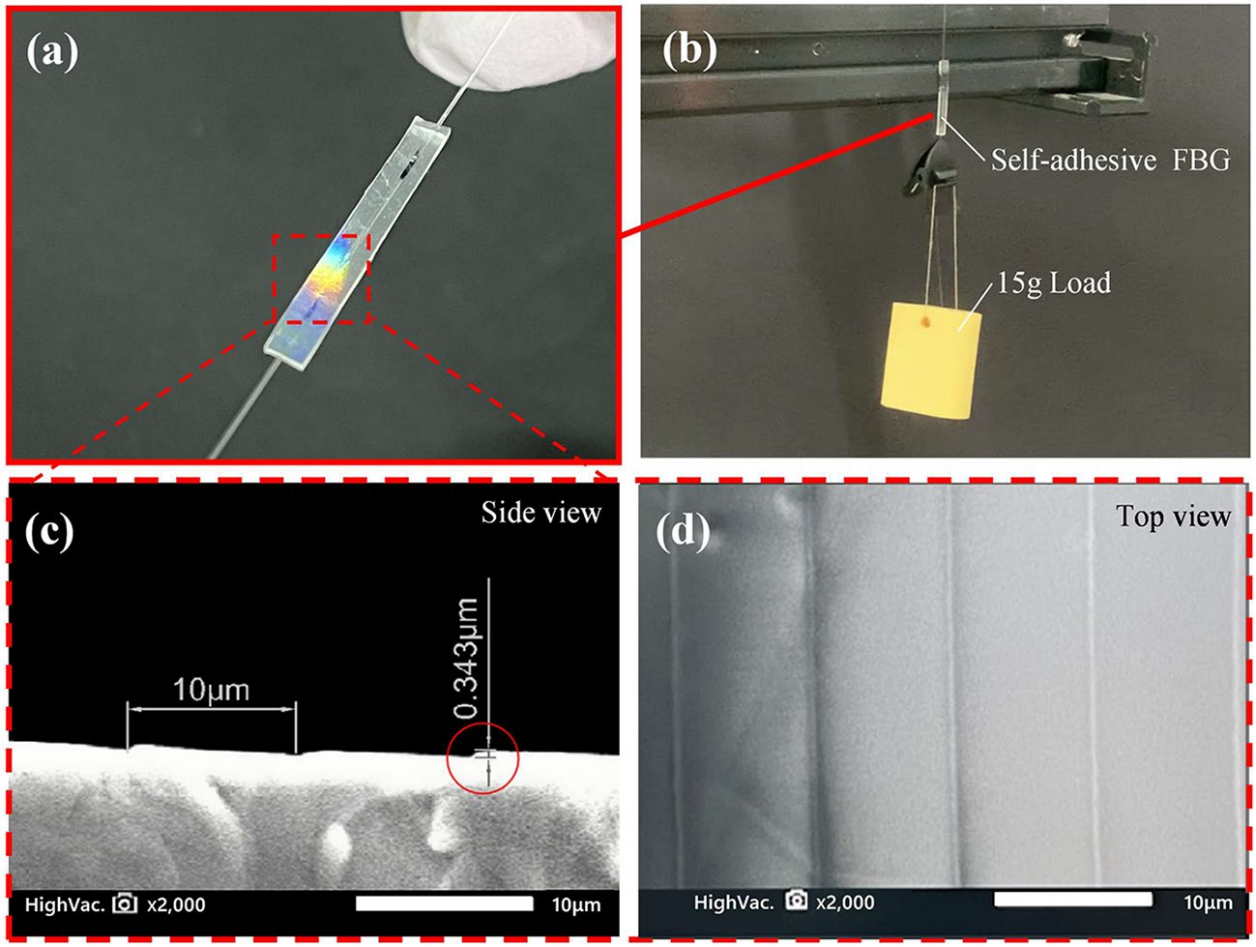
(**a**) Photo of the manufactured sensor sample. (**b**) Self-adhesive FBG with a rubber hanging. **(c)** SEM image of self-adhesive FBG cross-section. (**d**) SEM image of self-adhesive FBG surface.

A sample of the manufactured self-adhesive FBG sensor is shown in Fig. 4a. In the figure it is possible to observe light diffraction on one of the packaging surfaces, indicating the self-adhesive side. A first simple verification of the self-adhesion property of the manufactured PDMS-packaged FBG sensor is carried out by hanging a rubber from the packaging, while this is self-attached to an aluminium bar without the use of any glue. The bar has a smooth aluminium surface with black oxide film on it. Figure 4b shows a picture of the test demonstrating the strong self-adhesion property provided by sample, which allows hanging a rubber of 15 g with no problem.

To verify the microarray adhesive structure of the manufactured PDMS packaging, images obtained by a scanning electron microscope (SEM) are shown in Fig. 4c and d. SEM images show that the microarray structure is wedge-shaped, neatly arranged and evenly distributed, with a period of 10 μm and a depth of 0.343 μm, as designed. The images demonstrate that the designed microarray structure of the diffraction grating has been successfully imprinted in the PDMS surface. Since the peak and valley depth of the microstructure surface are nanometre-to-micrometre scale, the wedge-shaped microstructure could easily attach to any kind of smooth surface, increasing the contact area and improving the adhesion force. When a lateral force is applied (in a parallel direction compared to the contact surface), the wedge-shaped microstructure wraps the wave peaks and meshes with each other. This way, the wedge-shaped microstructure can support large tangential forces, as will be tested in the next section.

## Results and discussion

### Evaluation of self-adhesion force

The dry adhesion capability of the microarray self-adhesive structure is evaluated by monitoring the tangential adhesion force that the packaged FBG sensor can hold before detachment. For this, several wedge-shaped microstructure samples are attached to four different holding materials: silicon (Si) wafer, polymethyl methacrylate (PMMA), aluminium, and carbon fibre-reinforced polymer (CFRP). Note that these four holding materials have been selected to prove the wide application scope of the proposed self-adhesive FBG. They are representative of semiconductor materials, plastic materials, metal materials and composite materials, which are widely used in construction, energy, transportation, electronic components, and many other fields, allowing us to test and verify a wide range of potential applications. The roughness of the four holding materials is 0.5 nm, 0.1 μm, 0.4 μm and 1.6 μm for the silicon wafer, plexiglass, aluminium and CFRP, respectively. Note that the silicon wafer surface is polished by a CMP (Chemical–Mechanical Planarization) process, so it has an extremely smooth planar surface compared to the other materials. The size of the self-adhesive PDMS-packaged FBG samples is 8 mm × 2 mm × 0.5 mm, with an elastic modulus of about 2 MPa.

**Figure 5:**
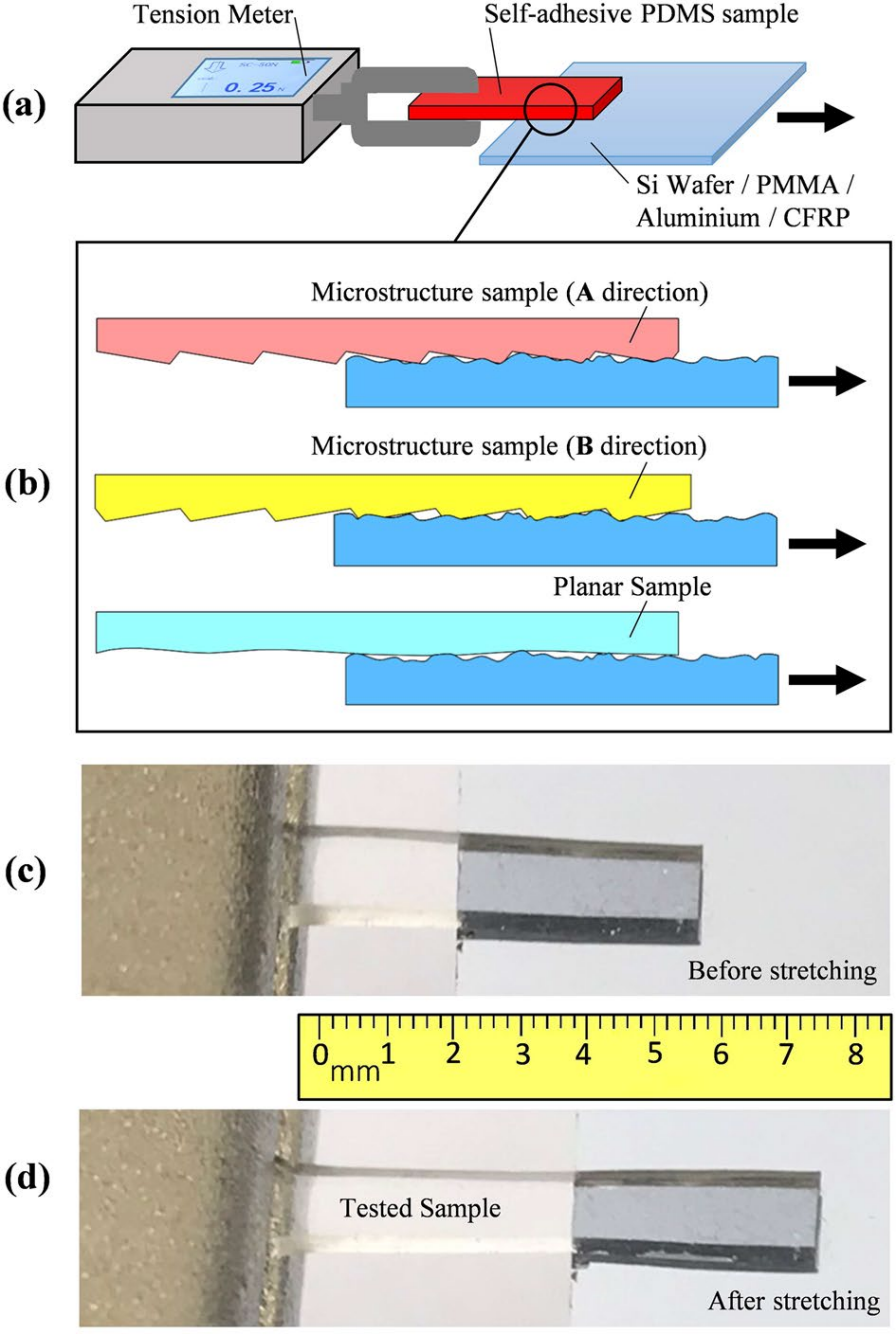
Experimental evaluation of the tangential adhesion force provided by the manufactured self-adhesive PDMS packaging. (**a**) Experimental setup. (**b**) Definition of pulling directions depending on the microstructure orientation. (**c**) Photo of self-adhesive sample before tangential force is applied. (**d**) Photo of self-adhesive sample after tangential force is applied.

To measure the adhesion force of a sample in each situation, half of the sample is attached to the holding material, providing a contact area of 4 mm × 2 mm, as shown in Fig. 5a. The other half of the sample is clamped to a tension meter, which applies horizontal tension to the sample until the bonded half of the sample is detached from the holding material. The force required for detaching the sample is recorded, and then compared for different situations. The experimental procedure to evaluate the adhesion force can be observed in Supplementary video 1.

Because the fabricated wedge-shaped microarray structure has directionality, the samples have been pulled in two different directions. The pulling direction of the wedge-shaped long side is defined as direction A, as depicted in the top case of Fig. 5b. The sample has also been pulled in the opposite direction (direction B), as illustrated in the second case of Fig. 5b. The adhesion forces in the two directions are then compared to the tensile force provided by a planar (flat) sample without the microarray structure on the surface (depicted by the last case of Fig. 5b), which can weakly bond to different holding materials due to the stickiness of PDMS. Figure 5c and d show the stretching and self-adhesive capabilities of the PDMS sample when a moderate lateral force is applied. No displacement of the attached sample section is observed in this case, as the edge of the sample does not slip while applying lateral force. When the applied tangential force remains below the maximum tolerable, then the PDMS packaging can provide excellent dry adhesion capabilities, supporting even dynamic tensions being periodically applied to the packaging, as can be visualised in Supplementary video 2.

**Figure 6:**
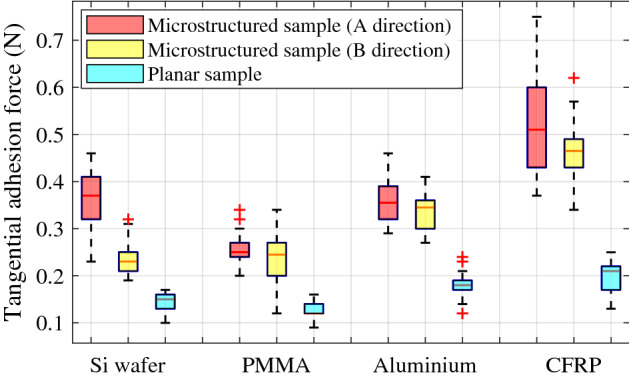
Tangential adhesion force of samples self-attached to different holding materials, for two pulling directions using microstructured packaging and using a planar packaging. Boxplots show the statistical distribution of the measured force resulting from 30 tests performed for each of the analysed conditions.

Figure 6 shows boxplots depicting the distribution of the tangential force that has been measured in 30 tests for each of the holding (monitored) materials. Results point out that the tangential forces in directions A and B are similar for a given material, whilst these forces are always higher than the ones measured using the planar packaging being weakly sticked to the monitored surface. It is worth noting that the adhesion force along direction A is in all cases slightly greater than that along direction B, although the overall difference is not very big. This is mainly due to the asymmetric wedge-shaped geometry of the PDMS adhesion surface microarray. Theoretically, when pulling along direction A, the long side of the serration acts so that the contact area is larger, leading to higher adhesion. On the contrary, when pulling along direction B, the short side of the serration acts, leading to slightly smaller adhesion. Note also that, on the micro scale, the actual adhesion is partially affected by the random local roughness of the contact surfaces, resulting in only minor differences of the adhesion forces existing in the two pulling directions. Results also verify that the average value of the measured tangential adhesion forces vary for different surface materials (see that CFRP shows a slightly higher adhesion force compared to the other materials). It is believed that these differences are essentially due to the distinct polarizabilities and polarities of the materials used in this experiment, which lead to different levels and types of van der Waals forces^20^, and therefore to different tangential adhesive forces.

### Temperature response of the sensor

**Figure 7:**
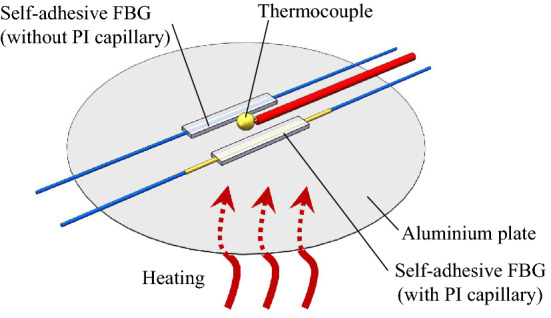
Experimental evaluation of the temperature response of the self-adhesive FBG sensors with and without PI capillary.

To verify the temperature response of the self-adhesive FBG sensing structure, samples in the two different packaging forms (i.e., one sample with and another without the PI capillary) have been attached to an aluminium plate, as shown in Fig. 7. In addition, a thermocouple is glued (using 502 cyanoacrylate glue) to the plate to provide reference temperature measurements.

**Figure 8:**
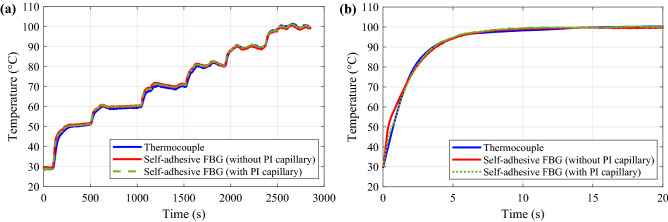
Comparison between the temperature measured by the self-adhesive FBG sensors with and without PI capillary and a reference thermocouple. Experiment performed applying (**a**) a stepwise temperature increase, and (**b**) a single 70 °C temperature step.

The temperature of the aluminium plate has been increased from room temperature (~ 30 ºC) up to ~ 97 ºC with steps of 10 ºC and held for 5 min until temperature stabilisation. Note however that to better visualise the thermal response time of the packaged FBG sensors, the first step was set to 20 ºC. Figure 8a shows the temperature evolution measured by the two self-adhesive PDMS-packaged FBG sensors (with and without PI capillary) and the standard thermocouple placed next to them. Results verify that the temperature evolutions measured by the two self-adhesive FBG sensors follow very closely the reference temperature given by the thermocouple. Comparing temperature values obtained after stabilisation, the packaged FBG sensors with PI capillary and without PI capillary show to have root-mean-square (RMS) errors of 0.44 ºC and 0.52 ºC, respectively; whereas the maximum absolute error resulted to be below 0.7 ºC.

To verify the temperature response speed of the two self-adhesive FBG sensors (with and without the PI capillary), a temperature step of 70 ºC has been applied to the aluminium plate. Figure 8b shows the temperature increase from 30 ºC up to 100 ºC measured by the two samples and the reference thermocouple. Results show that the two packaging forms of PDMS-packaged FBG sensors have similar response time compared to the thermocouple, validating the good thermal response of the PDMS packaging. The time required for the packaged FBG sensors and thermocouple to reach the final temperature is about 9 s, which corresponds to the time required for the measured temperature to vary 98% of the applied thermal change.

### Residual strain evaluation

The PDMS packaging proposed in this work isolates the embedded FBG from strain through the mismatch of the elastic modulus of materials. PDMS packaging actually acts as a flexible thin cushion that permits a large reduction of elastic modulus, from the high value of the monitored object to the low elastic modulus of PDMS. Consequently, the strain of the monitored structure will not be directly transferred to the FBG but absorbed by the PDMS packaging. This allows the FBG to maintain its original shape while the monitored structure undergoes large deformation, leading to much lower FBG strain compared to the strain affecting the monitored structure. At the same time, the packaging design considers a small gap between the PI capillary and the FBG sensor, allowing the FBG to move slightly inside the PI capillary when strain is applied; this helps to further mitigate the strain transferred to the packaged FBG sensor.

**Figure 9:**
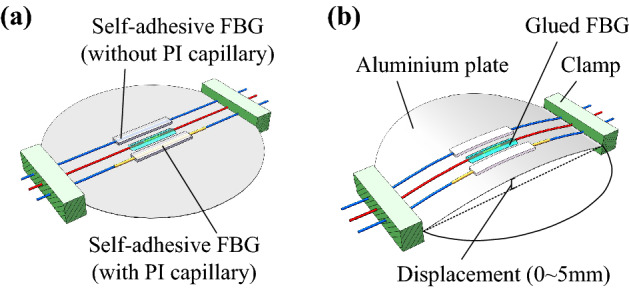
Characterisation of the residual strain transferred to the PDMS-packaged FBG sensors (with and without PI capillary). (**a**) Description of the experiment with an aluminium plate at an initial straight position, and (**b**) after bending the plate.

To verify this strain damping effect, the self-adhesive FBG sensors with and without PI capillary have been self-attached to an aluminium plate, as shown in Fig. 9a. In addition, a conventional unpackaged FBG sensor has been glued (using 502 cyanoacrylate glue) to the plate to measure the actual strain applied. The plate has been bent by applying a displacement in its central part, as shown in Fig. 9b, so that some strain is applied to the central section of the structure where the self-adhesive and reference FBG sensors are placed.

**Figure 10:**
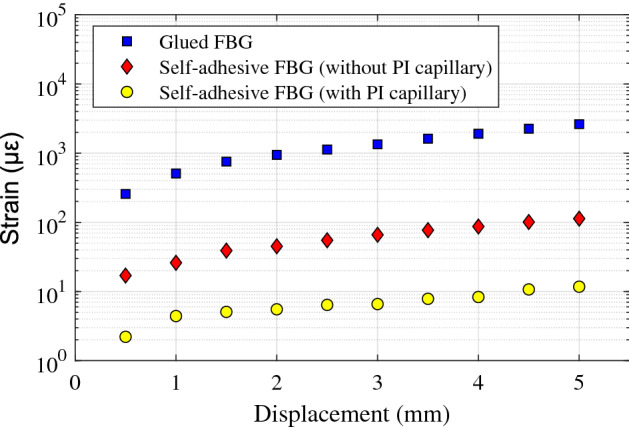
Comparison of the strain measured by the self-adhesive FBG sensors with and without PI capillary, and the reference strain obtained by a glued FBG sensor.

Figure 10 shows a comparison of the strain values obtained as a function of the applied vertical displacement and measured simultaneously by the two packaged self-adhesive FBG sensors and the glued FBG sensor. Results point out that the strain measured by the self-adhesive FBG without PI capillary packaging corresponds to approximately 4.9% (on average) of the strain measured by the glued FBG sensor. This represents a significant reduction of the strain transfer when compared to the transfer obtained with the glued FBG. For the PDMS-packaged FBG sensor containing the PI capillary, the residual strain measured by the sensor is further reduced, reaching only an average of 0.49% of the strain measured by the glued FBG. This represents a reduction of strain in a factor 200 approximately. The additional strain reduction is mainly provided by the narrow gap between the PI capillary and FBG sensor, securing a higher strain isolation capacity. These results match very well the obtained FEM results, validating the large mitigation of the strain measured by the proposed self-adhesive FBG sensor packaging with PI capillary, making it an ideal solution for temperature sensing with minimal impact from strain.

## Conclusion

In this work, a self-adhesive PDMS packaging for strain-free temperature monitoring based on FBG sensors has been proposed, fabricated and tested. The proposed low-cost packaging has been manufactured with a soft material, like PDMS, which causes the mismatch of elastic modulus between the optical fibre and the surface of the bonding object, enabling the absorption of applied strain in order to mitigate its impact on FBG-based temperature sensing. Further strain mitigation is achieved by using a polyamide capillary inside the PDMS packaging where the FBG sensor is embedded. The packaging is fabricated with wedge-shaped microstructures on one of the surfaces, which permit clean, dry adhesion to different type of materials using van de Waals forces. In addition to protecting the optical fibre and embedded sensor, the proposed packaging gives FBG sensors the capability to be attached to any macroscopic smooth surface with a dry (clean) adhesive and to be easily removed leaving no residuals in the monitored structure, as illustrated in Supplementary video 3.

The experimental tests and results have demonstrated that the self-adhesive FBG sensor has a strong dry adhesion and can significantly mitigate the effect of strain on accurate temperature measurements. It is worth noting that the adhesion to surfaces with different roughness will be slightly different depending on the material, which needs to be distinguished in future use. Specific shape and depth designs of the microstructures might be required to optimise adhesion to particular types of surfaces. Currently the manufactured samples used in this first study have been tested in a wide temperature range reaching up to about 100ºC. Preliminary observations have indicated that the tangential adhesion force might be reduced with increased temperatures, presumably due to thermal expansion of the PDMS packaging, which changes the contact conditions of the microstructures providing van der Waals adhesion forces. Note also that the working temperature range of PDMS is from – 55 ºC up to about 200 ºC. This corresponds to a range where there are no obvious changes in the material properties, which means that PDMS can remain soft with a low elastic modulus. Therefore, the PDMS packaging can still be effectively used as a strain isolation layer when the temperature changes within the specified range. Further studies on the material, microstructures, and their array layout are still required to enhance the thermal response of the packaging adhesion force, and to extend the features of the packaging to much higher temperature ranges.

The self-adhesive bonding capability of PDMS-packaged FBG sensors to different materials for various potential applications can be observed in Supplementary video 4. It is believed that the dry adhesion provided to FBG sensors can be of great interest in applications where clean and non-invasive monitoring is required, thus avoiding the need of glues or resins to paste the FBG sensors. The capability of removing the packaged FBG sensor is expected also to find several potential applications where the reusability of the FBGs could bring many advantages, either because the low cost and/or to perform quick measurements without the need of using glues.

## Supplementary Information

Supplementary Video 1.

Supplementary Video 2.

Supplementary Video 3.

Supplementary Video 4.

## Data Availability

The data obtained and analysed in this study are available from the corresponding author upon reasonable request.
